# Treatment of Frequent or Chronic Primary Headaches in Children and Adolescents: Focus on Acupuncture

**DOI:** 10.3390/children10101626

**Published:** 2023-09-29

**Authors:** Ilaria Bonemazzi, Magherita Nosadini, Maria Federica Pelizza, Chiara Paolin, Elena Cavaliere, Stefano Sartori, Irene Toldo

**Affiliations:** Juvenile Headache Center, Department of Woman’s and Child’s Health, University Hospital of Padua, 35128 Padua, Italy; ilaria.bonemazzi@aopd.veneto.it (I.B.); margherita.nosadini@aopd.veneto.it (M.N.); mariafederica.pelizza@aopd.veneto.it (M.F.P.); chiara.paolin@aopd.veneto.it (C.P.); elena.cavaliere@aopd.veneto.it (E.C.); stefano.sartori@unipd.it (S.S.)

**Keywords:** acupuncture, chronic headache, treatment, children, adolescents, alternative medicine, complementary medicine, CAM

## Abstract

Background: Acupuncture is a spreading and promising intervention, which has proven to be very useful in the treatment and prevention of chronic pain, in particular chronic headaches, in adults; the literature about the treatment of pediatric chronic headaches is scarce. In addition, few guidelines advise its use in children. The aim of this review is to collect all relevant studies with available data about the use, effect, and tolerability of acupuncture as a treatment for pediatric primary headaches. Methods: This is a narrative review based on eight studies selected from 135 papers including pediatric cases treated with acupuncture for headache. Results: Despite the differences in tools, procedures, and application sites, acupuncture demonstrated a positive effect on both the frequency and intensity of headaches and was well tolerated. There are no studies considering the long-term efficacy of acupuncture. Conclusion: Further additional studies are needed on acupuncture in children and adolescents, with larger series and standardized procedures, in order to better assess efficacy, tolerability, and long-term prognosis and to define guidelines for the use of this promising and safe treatment. It is particularly relevant to identify safe and well-tolerated treatment options in pediatric patients affected by recurrent and debilitating headaches.

## 1. Introduction

The use of complementary and alternative medicine (CAM) therapies has increased in the United States since 1990, and, in recent decades, has also been spreading in Europe [1,2]. Acupuncture is one of the most used CAM therapies [3,4] and, in the last few years, it has also been recognized as an important integrative medicine [5,6,7]. This ancient procedure, in recent years, has been recognized by the National Institutes of Health (NIH), the Food and Drug Administration (FDA), and the World Health Organization (WHO) for the treatment of different kinds of chronic pain [8,9,10]. With the spreading and improvement of acupuncture, over the years, acupuncture-related therapies have emerged which are alternatives to the use of needles, including those that utilize electrical stimulation, heat, and magnets [11].

Concerning acupuncture as a headache treatment, there are many studies that consider its utility, efficacy, and tolerability in adult patients, as both an acute and a preventive intervention [8,12,13,14]. Guidelines and systematic reviews recommend its use for headache (especially migraine) prevention, particularly in children, to avoid drug side effects [15,16,17,18,19,20,21,22].

Treatment regimens for chronic headache patients can be expensive, requiring intravenous access and hospitalization of the patient [12,23]. In addition, studies indicate that treatment response tends to decrease in children with chronic headache disorders [24,25], and recurrences of migraine following acute intravenous treatment are common [26,27]. Nevertheless, the use of acupuncture in children with headaches is limited, as it is so far reserved as an add-on therapy [3,28], a third-line therapy [22], or a preventive therapy [28], for several reasons, summarized in paragraph 5.

Since the literature is very limited and there are no standardized and globally recognized protocols and procedures for acupuncture in children with headache [27], the aim of this review is to collect all relevant studies with available data about the use, efficacy, and tolerability of acupuncture in the treatment of pediatric headaches.

## 2. Definitions and Epidemiological Data

### 2.1. Chronic Headaches in Children and Adolescents

The average incidence of headaches in the population younger than 20 years ranges from 5.9 to 58.4%, most of which are primary headaches [12,29,30]. Tension-type headache (TTH; 4.5–29%) and migraine (M; 10.1–58.0%) are the most frequent types of primary headaches for children and adolescents [6,7,31,32,33,34,35].

In many cases, headache can have a large impact on children’s quality of life [29], can persist into adulthood (in up to 73% of children), and, in many cases, can even evolve into chronic conditions [29,36,37]. Children with chronic headaches constitute the most debilitated subgroup of the headache population; therefore, these disorders require specialized management [38].

The definitions and diagnostic criteria of chronic headaches, according to the International Classification of Headache Disorders (ICHD-3) [39], are reported respectively in Table 1 for chronic migraine (CM), and in Table 2 for chronic tension-type headache (CTTH).

### 2.2. Acupuncture

Acupuncture is an old practice from ancient Chinese culture, the existence of which was tracked in documents dating back to 500 BC [8].

Nevertheless, acupuncture started to spread to Europe slowly, only many years later, with studies that confirmed the effectiveness of somatic and auricular acupuncture regarding pain control [14,40,41,42]. Among the first utilizations in Europe, it is notorious in the work of Paul Nogier, who established the map or homunculus of ear points [43].

After a workshop on its efficacy and safety, acupuncture was recognized as a “medical device” (class II) in 1996 by the NIH and the FDA [8,9].

In 1997, a consensus meeting was conducted, where it was evidenced that acupuncture was effective for many medical conditions in adults, such as postoperative pain, chemotherapy-related nausea and vomiting, postoperative dental pain, dysmenorrhea, stroke rehabilitation, tennis elbow, headache, fibromyalgia, myofascial pain, osteoarthritis, carpal tunnel syndrome, low back pain, poststroke rehabilitation, tendonitis, and asthma [44].

Since then, the use of acupuncture has been reported as effective in controlled clinical trials for many other types of painful conditions, including up to a total of 28 attested conditions in 2003, according to the 2003 WHO publication [9,10,45,46,47].

Currently, acupuncture is one of the CAM therapies most frequently recommended by internal medicine and family doctors [4,48,49], with reported 1:10,000 to 1:100,000 incidence of serious side effects when performed by a licensed acupuncturist [4,50,51,52].

Especially in children, guidelines and systematic reviews recommend the use of acupuncture for headache (in particular migraine) prophylaxis [15,16,17,18,19,20,21].

In particular, the National Institute for Health and Care Excellence advises considering a course of up to 10 sessions of acupuncture over five to eight weeks for the prophylactic treatment of CTTH, and for the third-/fourth-line treatment of CM [22].

#### How Does It Work?

Experimental measurable and repeatable acupuncture clinical effects are reported. There are several hypotheses for the mechanism of the control of pain perception [53,54] and the anti-inflammatory effects [55,56,57,58]. Activation of descending inhibitory pain control systems has also been suggested, but the exact mechanism is not fully known [34,59].

One theory explaining how acupuncture modulates pain is the neurohumoral theory. It is believed that the analgesic properties of acupuncture are mediated, in part, by a cascade of peptides such as enkephalin, endorphins, and monoamines, which are activated by stimulation of acupuncture points and create a sensation of “de qi” (a feeling of fullness, heaviness, pain) [9,60].

The stimulation of acupuncture points leads to the stimulation of A delta fibers with the activation of the anterolateral tract of the spinal cord, midbrain periaqueductal gray, and raphe nucleus, which leads to the release of the inhibitory peptides, norepinephrine, and serotonin in the spinal cord. β-endorphins and corticotropin are released by stimulation of the hypothalamic–pituitary axis [9,61].

Some studies have recorded quantitative changes in panopioid activity and β3-endorphin levels in the blood and cerebrospinal fluid (CSF) of migraine patients: one of them [62] showed a decrease in patients’ plasma β3-endorphin levels during episodes but not during periods without migraine, and two others [63,64] demonstrated a decrease in β3-endorphin in the CSF. Another interesting study demonstrated higher levels of β3-endorphins in migraine patients as compared with control patients [65]. In other studies, 13-endorphins were used as a marker for assessing the influence of nonpharmacologic treatment methods in patients with migraine. An increase in the level of β3-endorphins in plasma, as compared with pretreatment levels and those of a control group, was observed in patients with migraine who were treated with electroacupuncture [66,67].

Pomeranz et al. supported the role of endogenous opioids by demonstrating the reversal of acupuncture analgesia after the administration of naloxone, an opioid antagonist [68]. In support of this hypothesis, Pintov et al. found an association between a significant reduction in migraines and a β-endorphin decrease [67]. Han et al., in addition, found that low-frequency (2–10 Hz) electrical stimulation of acupuncture points leads to increased endorphin release, while high-frequency stimulation (100 Hz) leads to increased dynorphin release [69].

Another hypothesized mechanism for acupuncture analgesia is that acupuncture stimulates polymodal receptors [70].

In some functional magnetic resonance imaging (fMRI) and positron emission tomography (PET) studies, the stimulation of specific acupuncture points on the body was related to the deactivation or activation of corresponding areas of the brain [71,72,73,74]. Some fMRI studies have shown, in addition, changes in brain blood flow following acupuncture treatment, and normalization of activity in areas of the limbic system, as well as areas of the “pain matrix” [12,41,45,47,75,76,77].

It seems that acupuncture also provokes the release of analgesic peptides like cholecystokinin octapeptide, and its antisense RNA increases the analgesic effects [78,79].

Other studies described local changes at acupuncture points in needle placement, such as connective tissue changes [80] and changes in electrical resistance [81]. Those effects were related to clinical responses in adolescent girls with pelvic pain [47]. It appears that laser–tissue interactions induce inhibition of Na+K+ATPase, affecting the resting potential of cells. In addition, laser can cause the reversible blockade of mitochondrial transport, resulting in disruption of neurotransmission in A delta and C fibers and subsequent pain relief [82].

Some other acupuncture systemic effects are reported, such as acceleration of nerve generation [83], neuroplasticity [84], changes in central and peripheral blood flow regulation [85], and alterations in immune function [86].

Concerning auricular acupuncture in the treatment or prevention of headaches, it is based on the fact that the ear receives innervation from the auricular branch of the vagus nerve, the auriculotemporal branch of the trigeminal nerve, the great auricular nerve from the second and third cervical roots, and the facial and glossopharyngeal nerves [87]. The activation of the trigeminal–vestibulocochlear reflex and the spread of neurogenic inflammation could be the causes of vestibular migraine symptoms [88,89]. Auricular acupuncture could act via the trigeminal innervation of the ear in the treatment of migraine. It is known that the ventromedial medulla oblongata, ventrolateral periaqueductal gray, locus coeruleus, and raphe magnus nucleus, structures that modulate trigeminal nociceptive input, are affected by acupuncture [12,71,90,91,92,93]. In addition, the ear is provided with neurovascular complexes, which contain capillaries, lymphatic vessels, and myelinated and unmyelinated nerve fibers [87]. The ear also has cholinergic and adrenergic fibers, which can cause neurotransmitter release [12].

## 3. Materials and Methods

PubMed, Cochrane, and Mendeley were used for the search, with the following queries being entered: “acupuncture headache”, “CAM headache”, and “complementary and alternative medicine headache”. The search was filtered for papers that included children and adolescents (age range 0–18 years).

A total of 135 full-text articles were evaluated for eligibility. Articles written in languages other than English were excluded (*n* = 22). For seven papers, full texts could not be found. Fifty-three articles, reporting data different from those of interest, were excluded. From the remaining 53 studies, narrative reviews (*n* = 8), studies in an adult population (*n* = 32), studies with fewer than 3 cases (*n* = 3), and studies with insufficient data (*n* = 2) were excluded.

Finally, 8 studies based on exclusively pediatric (*n* = 6) and mixed (children and young adults up to 21 years of age; *n* = 2) series published between 1997 and 2021 were included. These 8 works were analyzed, with the collection of the following data: demographics, type of headaches, evaluation of the pain intensity, procedures of acupuncture, site of insertion, combination with other treatments, efficacy, and adverse effects.

Most of these studies are retrospective; therefore, to collect clinical data, they were based on questionnaires [4,94], semi-structured interviews [28], or clinical interviews [1,27,94] about the headache histories of patients. One study is a prospective interventional study [12], and two of them are case–control studies [67,95]. The two case–control studies are randomized, one double-blinded [95] and the other single-blinded [67]; in both cases, there are no significant differences in age, gender, headache type, baseline headache frequency, intensity, or duration of headache attacks between the active and the placebo groups [67,95].

A narrative synthesis of the included studies was then conducted, as the available data did not allow for the performance of a meaningful meta-analysis.

## 4. Population

The study population found was mostly composed of pediatric and adolescent patients with ages ranging from 0 to 18 years [12,27,28,67,94,95], except in two studies in which the upper limit was up to 21 years [1,4]; the patients were mostly female (from 58% [67] to 89% [12]).

The patients were all headache sufferers; the main diagnoses, conducted based on ICHD criteria [39], were migraine (M) (50–82%) [4,28,95] and tension-type headache (TTH) (18–50%) [28,95], with chronic forms often present [12,27]. The duration of headache history ranged from 1 to 9 years [27,95], but, in most cases, was not defined [1,12,28,67,94].

In some studies, no prior prophylactic treatment was given [12,67,95]; in others, these data were not specified [1,4]. In one study, patients had undergone other prophylactic treatments in the past before acupuncture, with variable or unspecified effects [27]; in another one, acupuncture was used in addition to conventional treatments [28].

The main clinical features of the patient populations are summarized in Table 3.

## 5. Why Do Patients Choose Acupuncture Treatment?

Considering also a study with a mixed adult and child population, acupuncture is among the most widely used CAM therapies (up to 58.3%), followed by massage (46.1%) and relaxation techniques (42.4); other CAM therapies include thermotherapies, diet, music therapy, psychophonia, climatotherapy, use of high-dose megavitamins or herbal medicine, and others [3,4].

The choice to use a CAM method instead of a traditional one seems to be due to the wish to avoid chronic use of drugs with their related side effects (70% of patients), the desire for an integrated approach (52%), the reported dissatisfaction with current conventional medicine (32%), or a more suitable youth disposition (20%) [28]. However, it is known that most patients (up to 71.1%) use CAM in addition to their conventional treatments, as integrative medicine [28].

Focusing on acupuncture, many studies reported some dropouts due to the children’s or children’s parents’ fear of needles [1,4,12]. However, Zeltzer et al. found that no patients discontinued treatment because of fear or anxiety about the procedure and that anticipatory anxiety assessment scores were low. This might suggest that patients rated the treatment as easily tolerable and not a source of anxiety [94]. A good alternative procedure to standard acupuncture is laser acupuncture, which is nontraumatic, and could be applied to children of all ages because it is less disturbing, regardless of platelet count and coagulation status [95].

Most patients experience acupuncture as a preventive treatment (80% of patients), and a few for acute “on demand” therapy (5%), or for both (15%) [28].

Interestingly, acupuncture is often used not directly for headaches but to reduce stress, which seems to contribute to the recurrence and maintenance of migraine [28].

Predictive factors for choosing CAM use appear to be female sex, younger age, higher parental education level, and healthy lifestyle [28].

## 6. Procedures

### 6.1. Acupuncture Tools

Some authors utilized Aiguille D’Acupunture Semi-Permanente (ASP) needles [1,12]. Gottschling et al. used an acupuncture laser: Modulas-Handy (2/99, 30 mW, 830 nm, continuous wave, power density 3.8 W/cm^2^, 1 mm laser beam diameter) [95].

One study used different acupuncture techniques: basic acupuncture, electroacupuncture (EA), auricular acupuncture, laser acupuncture and electron transfer facilitated by ionic cords, and moxibustion [1]. Acupuncture with needles was performed with DBC Spring Ten needles (ranging in length from 15 mm to 50 mm), EA with a HAN E600 electroacupuncture unit, auricular acupuncture with ASP, moxibustion with Ultra-Pure “Gold Mountain” Moxa in hand-rolled cones, electron transfer with Manaka ion pump cables, and laser therapy with an HY05-A Aini therapeutic laser (the large laser probe—about 4 cm in diameter—contains a central laser with a power of 300 mW and a wavelength of 810 nm, seven laser output holes, and six semiconductor laser output holes with a power of 5 mW and a wavelength of 650 nm, at a distance of 5 cm from the patient’s face; the small laser probe has a power of 200 mW and wavelength of 810 nm and has a radius of about 3 mm) [1].

### 6.2. Acupuncture Protocols

In the case–control study of Pintov et al., a group was treated according to the principles of traditional Chinese medicine, with the acupuncture needle inserted subdermally, and another group (the control group) was treated with a needle of the same size inserted in the stratum corneum. Each child attended 10 weekly sessions of acupuncture treatments of 15 min [67].

In the Gottschling case–control study, a combination of traditional Chinese body acupuncture and auriculotherapy was applied, with the use of laser acupuncture, once a week for four consecutive weeks [95].

In another work, different treatment methods were used: basic needle acupuncture (165/174 patients), with three needle applications per visit (10–20 min for each application); EA (1/174), with both low-frequency and high-frequency stimulation (switching between 2 Hz and 100 Hz every 3 s); auricular acupuncture (4/174); laser acupuncture (1/174), with a large laser (at 28 J/cm^2^ energy, in continuous mode for 20 min per application) and a small laser (at 85 J/cm^2^ energy, in continuous mode, for stimulation of individual points for 30 s on each point); moxibustion (1/174) (eight times for each point where moxibustion was performed); and electron transfer facilitated by ionic cords (2/174) [1]. The latter technique is practiced by placing alligator clips on the skin at the points chosen for treatment and securing them with aluminum tape; the unidirectional transfer of electrons, from the black clip to the red clip, is facilitated by a germanium diode (for 20 min per treatment session) [1].

Also, the study of Kemper included different acupuncture types: intradermal needle acupuncture (45%), moxibustion or heat (85%), cupping (26%), and magnets (26%). Many children received >1 type of point stimulation. The median number of acupuncture treatments was eight, in almost 3 months [4].

Graff et al. practiced auricular acupuncture, with a mean number of needles of two and a maximum number of four, two in each ear [12].

### 6.3. Application Sites

In some studies, acupuncture points were chosen according to universally recognized traditional Chinese medicine (TCM) criteria, and individually for each patient [94]. Some authors utilized methods to locate efficacious ear points, for example, a needle contact test and/or an electrical point finder which emits an acoustic alarm when a change in electrical resistance is detected, signifying a potential active auricular acupoint [1,12]. A similar procedure, with a computer-based measurement of skin resistance differences, was applied in the Gottschling case–control study [95]. In this study, the basic points for patients with frontal headaches were the large intestine and stomach; for patients with lateral headaches this point was the gallbladder, and for patients with occipital pain these points were the small intestine and bladder [95]. Additional body and ear acupuncture points were chosen individually; a mean of 8.4 (±4.7) acupuncture points per patient and session were stimulated [95].

Another protocol included the insertion of three acupuncture needles in the upper and lower extremities [67].

### 6.4. Combination with Other Treatments

Zeltzer et al. combined acupuncture treatment with hypnosis in order to make the procedure more readily acceptable by the younger age group (6 years old) and to facilitate the initial tolerability of acupuncture in needle placement. As regards the type of hypnosis used, as there were no universally accepted definitions, a procedure involving progressive muscle relaxation and guided imagery was followed [94]. The treatment sessions (six in total) were conducted once a week for 6 weeks, for about half an hour each [94].

## 7. Acupuncture Effect

### 7.1. Assessment of Acupuncture Effect

Headache intensity ratings were recorded in most of the studies as pretreatment and post-treatment pain scores [1,12,27,67,95]. Pain scores from 0 to 10 based on a numerical self-reported visual analog pain score (VAS) were recorded in most cases [1,12,67,94,95]. In one work, an 11-point numeric rating pain scale was utilized [27]. Pintov et al. also determined endorphin levels in plasma, and because the binding of diprenorphine is affected by the plasma concentration, corrected the values obtained according to a standard curve of plasma devoid of opioid peptides (to which no protease inhibitors were added). The panopioid activity was calculated as % of inhibition (inhibition of binding by plasma) [67].

In addition to the VAS score, Zeltzer et al. assessed, through the Varni–Thompson Pediatric Pain Questionnaire (V-T PPQ), how effective parents felt acupuncture and hypnosis were in reducing their child’s pain. This questionnaire considers average pain in the previous week and current pain, including an index of disturbance of functioning in certain areas, specifically general physical activity, sleep, appetite, ability to perform schoolwork, activities with friends, activities at home, anger, sadness, and nervousness [94].

In one study, the Children’s Depression Inventory (CDI) was also considered to record symptoms of depression, and the State-Trait Anxiety Inventory for Children (STAIC)—State Version was used to assess anxiety [94].

### 7.2. Effect of Acupuncture on Headaches

The use of acupuncture in each study as acute or preventive treatment is summarized in Table 4.

Self-reported perceived benefits regarding headache were described in 57% of the cases [28].

Considering both parents’ and children’s opinions about their experience with acupuncture for headaches, 67% of patients and 60% of parents reported a positive experience, while only a few patients and parents felt it had made no difference, and one parent reported that the child’s pain seemed worse after the treatment [4]. There was a significant reduction in children’s and parents’ ratings of current pain from before to after treatment (the average rate went from 3.46 to 1.93 for children and from 3.19 to 1.81 for parents), and there was also a reduction in children’s average pain ratings in the week after treatment compared with the week before (from 3.86 to 2.79 for children and from 3.33 to 2.93 for parents) [94].

Acupuncture provided by a board-certified medical acupuncturist significantly decreased, in a few minutes, headache intensity, with a mean change (up to seven points on the VAS scale [12]) [12,27].

In the Graff et al. study, 63.7% (14/19) patients had complete resolution of their migraines after auricular needle placement [12].

Changes in pain intensity scores from pre- to postintervention were not significantly affected by age, gender, years lived with headaches, headache frequency, past visits, past therapies trialed, or school days missed [27].

In the Pintov et al. study, the group undergoing the treatment had a statistically significant clinical reduction in both the frequency (9.3 ± 1.6 per month vs. 1.4 ± 0.6) and the intensity (8.7 ± 0.4 vs. 3.3 ± 1.0) of migraine headaches before and after treatment [67], while frequency (9.4 ± 1.5 vs. 9.3 ± 1.4) and intensity (7.8 ± 0.6 vs. 6.2 ± 0.4) did not change in the control group [67]. In addition, no significant change in the panopioid activity of the plasma in the control group was evident when comparing values from before and after the treatment, while a significant potentiation of panopioid activity was found in the treated group (an increase of about 50%) [67]. There was no linear correlation between the decrease in the frequency of headaches and associated changes in panopioid activity [67].

Similar results were found in another case–control trial with laser acupuncture [95]: The mean improvement in headache frequency was significantly greater in the treatment group (the number of days with headache decreased by 7.0 days from the baseline to weeks 5–8) than in the placebo group, in which the decrease in headache frequency was not significant compared with the baseline (only 1.2 days) [95]. The headache frequency in the placebo group reached the baseline level in weeks 9–12; in the active acupuncture group, it instead stayed low until the study endpoint [95]. Headache intensity in the active treatment group decreased significantly compared with the baseline, and it was greater than in the placebo group [95]. Nevertheless, there was a significant beneficial effect of placebo laser acupuncture in weeks 1–4 and 13–16 from the baseline [95]. Another study found an average initial pain VAS of 5.5 vs. an average post-acupuncture pain VAS of 2.2, showing an improvement in pain VAS of 3.3 on a 10-point scale, which was statistically significant [1].

It was interesting to note that the daily headache duration on headache days did not change, while, due to a reduction in headache frequency, the monthly headache hours decreased [95].

Compared with the study results in adult patients, Gottschling et al. found that children seem to be more sensitive to acupuncture [95,96,97].

### 7.3. Effect of Acupuncture on Comorbidities

Considering the study by Zeltzer et al., child-reported depression symptoms remained unchanged from pre- to post-treatment, but it should be considered that more than 90% of patients did not have significant depression scores even before the start of treatment [94]. With regard to children’s anxiety, a trend of decreasing scores from before to after treatment was observed [94]. Even negative expectations based on parents’ previous acupuncture experiences did not impact treatment outcomes [94]. Along with the reduction in pain, there was a decrease in the interference of pain in the child’s functioning and activities as rated by both parents and children [67,94]. In particular, the scores for appetite, sleep, anger, sadness and nervousness, children’s difficulty with physical activity, activity with friends, household activities, and homework performance were lower after acupuncture treatment for both children and parents [67].

## 8. Adverse Effects

No adverse events or side effects were described in most of the studies [1,12,28,94,95], except in one case complaining of nausea [27]. Laser acupuncture has no side effects reported [95].

Nevertheless, in the literature other adverse effects in the pediatric population are reported: mild effects including crying, pain, bruising, transient hemorrhage at the puncture site, numbness at the puncture site, aggravation of preexisting symptoms, and vasovagal reactions such as dizziness or nausea/vomiting; moderate effects including severe bacterial infections at the site of needle insertion; and serious adverse effects including infections, pneumothorax, and nerve impairments [98].

## 9. Limitations

The main limitations of this study may be due in part to the methodology of the study itself, and in part to those of the studies being analyzed.

The first is due to the limited number of considered databases and to the inclusion of articles written only in the English language; therefore, additional studies could have been missed. However, PubMed, Cochrane, and Mendeley are among the largest available databases of medical studies.

Moreover, the available quantitative and qualitative data did not allow a meta-analytical study to be conducted; therefore, this narrative review, conducted using a retrospective observational research design, could present systematic and random errors.

Concerning the methods of the analyzed studies, many of them are retrospective chart reviews [1,4,27,28], and not all have a control arm [1,12,27,28,94]. Therefore, treatments were not compared with a control group, and because those responding to retrospective surveys may represent only a portion of headache sufferers, these surveys cannot be considered true efficacy data.

Not all authors specified the type of acupuncture and procedure considered [27,28]. In addition, the methods for the assessment of efficacy were different. When evaluating each intervention effect separately, some studies also could not separate out potential effects from other interventions that may have been administered at the same time in the same patient [1,27,94].

Some data would need to be expanded; in fact, many of these studies did not consider recurrence rates of headaches, or long-term prognosis [1,4,12,27,95]. Some studies also had a relatively low sample size [4,12,28,67,94,95].

Finally, some studies included pediatric patients with different kinds of chronic pain, not only headaches, without differentiating results [1,4,94].

## 10. Conclusions

In pediatric patients, it is necessary to avoid possible later disease chronicization and side effects of preventive treatments [28]. It is also necessary to find a treatment that decreases the need for hospitalization therapies, including intravenous and costly treatments, satisfying both the patients’ and the parents’ needs. Acupuncture may be an efficient alternative treatment for children with frequent or chronic primary headaches, due to its positive impact on headache frequency and intensity.

This nonpharmacologic treatment is well tolerated and safe, with no side effects in children and adolescents, as reported in the cited papers.

The available studies show encouraging results but have many limitations in methodology. Further additional studies on acupuncture in children and adolescents with primary headaches, with better selection of patients, standardization of procedures, and collection of clinical data, are needed in order to assess the best protocol and the best candidates for this treatment, its effect on headache and comorbidities, and its impact on long-term prognosis.

## Figures and Tables

**Table 1:** ICHD-3 diagnostic criteria of CM [39].

CM Diagnostic Criteria
Headache (migraine-like or tension-type-like) on ≥15 days/month for >3 months, and fulfilling criteria B and C Occurring in a patient who has had at least five attacks fulfilling criteria B–D for ^1^ MWA and/or criteria B and C for ^2^ MA On ≥8 days/month for >3 months, fulfilling any of the following: criteria C and D for MWA criteria B and C for MA believed by the patient to be migraine at onset and relieved by a triptan or ergot derivative Not better accounted for by another ICHD-3 diagnosis

^1^ Migraine without aura; ^2^ migraine with aura.

**Table 2:** ICHD-3 diagnostic criteria of CTTH [39].

CTTH Diagnostic Criteria
Headache occurring on ≥15 days/month on average for >3 months (≥180 days/year), fulfilling criteria B–D Lasting hours to days, or unremitting At least two of the following four characteristics: bilateral location pressing or tightening (non-pulsating) quality mild or moderate intensity not aggravated by routine physical activity Both of the following: no more than one of photophobia, phonophobia, or mild nausea neither moderate or severe nausea nor vomiting Not better accounted for by another ICHD-3 diagnosis

**Table 3:** Features of the patient populations in the literature.

Study	Patients (*n*)	Adults (A) /Children (C)	Age (Mean Age) (Years)	Sex (%)	Type of Headache (%)	Headache Duration (Years)
Dalla Libera et al., 2014 [28]	14	C	4–16 (12)	67 ^1^ f, 33 ^2^ m	70 ^3^ MWA, 18 ^4^ TTH, 12 ^5^ MA	-
Esparham et al., 2021 [27]	154	C	7–18 (14 ± 2)	79 f, 21 m	28 MWA, 23 ^6^ CMWA ^7^ MS, 23 MWA MS, 11 CMWA, 5 MA MS, 4 MA, 3 ^8^ NDPH, 1 ^9^ UH, 0.6 ^10^ A^11^ PTH, 0.6 ^12^ CPTH, 0.6 ^13^ UPTH	3.7 + 2.3
Graff et al., 2016 [12]	19	C	8–17 (14 ± 2.9)	89 f, 11 m	100 MS	-
Pintov et al., 1997 [67]	12 +10 ^14^ c	C	7–15 (9.8 ± 1.2) c: 10.4 ± 1.6	58 f, 42 m c: 60 f, 40 m	100 ^15^ M	-
McDonald et al., 2015 [1]	174	C(+A)	0–21 (13.9)	71.3 f, 28.7 m	-	-
Zeltzer et al., 2002 [94]	15	C	6–18 (13 ± 2.9)	-	100 M	-
Gottschling et al., 2008 [95]	22 +21 c	C	- (12.5 ± 2.8) c: 12.0 ± 2.4	63.6 f, 46.4 m 76 f, 23 m	50 M, 50 TTH c: 52.4 M, 47.6 TTH	^16^ s.w. 5.3 ± 3.7 ^17^ yo c: s.w. 3.9 ± 2.6 yo
Kemper et al., 2000 [4]	7	C(+A)	5–20 (16)	79 f, 21 m	100 M	-

^1^ f = female; ^2^ m = male; ^3^ MWA = migraine without aura; ^4^ TTH = tension-type headache; ^5^ MA = migraine with aura; ^6^ CMWA = chronic MWA; ^7^ MS = status migranosus; ^8^ NDPH = new daily persistent headache; ^9^ UH = unspecified headache; ^10^ A = acute; ^11^ PTH = post-traumatic headache; ^12^ CPTH = chronic PTH; ^13^ UPTH = unspecified PTH; ^14^ c = controls; ^15^ M = migraine; ^16^ s.w. = since when; ^17^ yo = years old.

**Table 4:** Acupuncture use: acute or preventive treatment.

Study	Patients (*n*)	Acute (A) or Preventive (P) Therapy
Dalla Libera et al., 2014 [28]	14	^1^ B (P 80%)
Esparham et al., 2021 [27]	154	A
Graff et al., 2016 [12]	19	A
Pintov et al., 1997 [67]	12 +10 ^2^ c	B
McDonald et al., 2015 [1]	174	A
Zeltzer et al., 2002 [94]	15	B
Gottschling et al., 2008 [95]	22 +21 c	B
Kemper et al., 2000 [4]	7	^3^ ns

^1^ B = both; ^2^ c = controls; ^3^ ns = not specified.

## Data Availability

The data of the current study (not included in this published article) are available from the corresponding author upon reasonable request.
